# Rebuilding to shape a better future: the role of young professionals in the public health workforce

**DOI:** 10.1186/s12960-021-00627-7

**Published:** 2021-07-13

**Authors:** Brian Li Han Wong, Ines Siepmann, Tara T. Chen, Shelby Fisher, Tobias S. Weitzel, Naomi L. Nathan, Diah S. Saminarsih

**Affiliations:** 1 Department of Population Science and Experimental Medicine, UCL Institute of Cardiovascular Science, Medical Research Council (MRC) Unit for Lifelong Health and Ageing at University College London (UCL), 5th Floor, 1-19 Torrington Place, Fitzrovia, London, WC1E 7HB UK; 2 Immunisation and Countermeasures Division, Public Health England, Colindale, London, UK; 3 Global Health Workforce Network (GHWN) Youth Hub, World Health Organization, Genève, Switzerland; 4 Faculty of Health, Medicine, and Life Sciences, Maastricht University, Maastricht, The Netherlands; 5 Department of Social Work, Tzu-Chi University, Hualien, Taiwan; 6 Department of Environmental and Occupational Health, École des Hautes Études en Santé Publique, Paris, France; 7 Department of Public Health, University of Copenhagen, Copenhagen, Denmark; 8 Institute of Public Health, Jagiellonian University, Krakow, Poland; 9 Office of the Director-General, World Health Organization, Avenue Appia 20, 1202 Genève, Switzerland

**Keywords:** Capacity building, Equity, Health workforce, Mentorship, Public health, Students and young professionals

## Abstract

The COVID-19 pandemic has made clear the extreme needs of the public health workforce. As societies discuss how to build up the capacity and infrastructure of their systems, it is crucial that young professionals are involved. Previous attempts to incorporate young professionals into the public health workforce have wrestled with inaccessibility, tokenisation, and a lack of mentorship, leading to a loss of potential workforce members and a non-representative workforce that reinforces systemic societal exclusion of diverse young people. These barriers must be addressed through robust mentorship structures, intentional recruitment and continuous support, as well as genuine recognition of the contributions of young professionals to build the sustainable, interdisciplinary, unified public health that is necessary for the future.

## Introduction

The coronavirus disease 2019 (COVID-19) pandemic has laid bare the limitations of existing public health structures around the world [1]. The resulting health and economic crises have further highlighted the need to build stronger public health infrastructures to fully meet the demands of public health in an equitable, innovative, and sustainable manner. Indeed, the pandemic has clearly identified one of the most untapped, overlooked resources of the current public health workforce (PHWF): its lack of integration of young professionals (YPs) [2]. While the workforce demanded additional resources, YPs existed in surplus. Unfortunately, infrastructure to adequately link them does not exist and YPs actively experience this frustrating disconnect on a daily basis.

YPs, defined here to be students and early career professionals, present great potential for the field of public health [3]. YPs can provide new perspectives and increase diversity, along with providing the necessary human resources the workforce so desperately needs [4]. The public health workforce itself has a constantly evolving definition; this article considers the PHWF to be anyone engaged in providing public health services [5]. While COVID-19 has made cracks in health systems more evident, it was already well-reported that public health needs improved, more sustainable infrastructure [6, 7]. The Youth Call for Action adopted at the Fourth Global Forum on Human Resources for Health called for policies to close the anticipated health worker gap, update health worker education systems to be equitable and interdisciplinary, and increase investment in the development of students and YPs [8]. YPs are an effective way to build workforce capacity and increase the representativeness of public health work; however, calls for inclusion of YPs have been either ineffective or have not been acted upon prior to and during COVID-19 [4, 9].

This commentary aims to address why the inclusion of YPs to date has been largely inadequate and unsuccessful. Drawing from the experiences of YPs in various academic and professional contexts, we shed light on the barriers faced by young public health professionals in hopes of catalysing the necessary transformations to shape public health to be representative, sustainable, effective, and dynamically adaptable to the world’s ever-changing population.

## Barriers to the integration of young professionals within the public health workforce

There is growing evidence that young professionals in the PHWF are facing unique challenges unlike previous generations. Many of these challenges have been further compounded by the COVID-19 pandemic, which has hindered YPs from launching their careers and transitioning from academic classroom settings to the workforce [2]. While the job-search process varies by region and sector, the realities of securing a position often lie in having a strong network, timing, having a strong voice within the public health community, and ultimately sheer luck. Inaccessibility, tokenisation, and a lack of mentorship have been identified as major barriers to successful integration of YPs within the PHWF [4]. Acknowledging and addressing these barriers will strengthen the PHWF through increased representativeness, diversified perspectives, and intergenerational learning, resulting in better health outcomes [10].

## Inaccessibility

Despite having highlighted how vital the PHWF is to the overall global economy, job prospects for YPs in public health remain unchanged, even amidst the current crisis [2]. YPs often find themselves either underqualified for entry-level positions or lacking professional public health certifications requiring years of experience, or overqualified for rudimentary positions which do not utilise their public health training. Concurrently, many public health hubs are located in cities with high costs of living that cannot be covered by entry level salaries, and internships and fellowships are often unpaid or underpaid [11]. Opportunities to gain experience in the workforce before seeking full-time employment are limited in number and often require unrealistic expectations of previous experience [9]. A significant number of innovative, grassroots public health initiatives (e.g. SDSN Youth and the Youth As Researchers global initiative on COVID-19) have been launched by YPs during the pandemic; however, nearly all were on a voluntary basis [12, 13]. This is not sustainable for YPs to grow their careers and perpetuates a homogenous form of public health only accessible to those with existing privileges and resources. In addition, YPs should not be limited to youth-run organisations; they should be included within existing public health entities. As such, there is much work to be done to address the current global challenges relating to both the unemployment and underemployment of YPs. A diverse and inclusive PHWF must involve the representation of professionals at all stages of their careers, in a manner that actively respects and reflects their individual experiences and values their contributions.

## Tokenisation

To date, existing forms of engaging and including YPs in the structure of organisations and/or the workforce have been primarily tokenistic in nature [14]. The participation and engagement policies of organisations and programmes are often top down, hierarchical, and lack transparency, with YPs having little if any real responsibility or voice in discussions and decision-making processes. This lack of trust is likely due to negative perceptions of YPs revolving around ageism, inexperience, and a limited desire to adapt existing norms [15]. Inclusion of YPs should also be inclusive and diverse, not limited to a single representative [16]. To ensure YPs are genuinely and not tokenistically engaged, they should be taken seriously and given real opportunities to direct/lead in each phase of projects: from concept development, to implementation, and through to evaluation [14]. The limitations of YPs do, of course, exist. To acknowledge these and limit further fragmentation of the PHWF, there needs to be positive and productive partnerships between YPs and more senior professionals [9]. The spaces, structures, and institutions in which YPs work must, therefore, actively recognise and support each of their unique talents, strengths, and limitations.

## Lack of mentorship

Successful YP engagement requires integration into the existing workforce. To achieve this, YPs expect a certain level of mentorship and support will be provided when they participate in new programmes and organisations. While mentorship opportunities may theoretically exist, a limited number of these involve the necessary in-depth engagement and instruction required for YP development. Peer mentorship, a strategy to evolve the understanding of public health by providing opportunities between young professionals, is also limited. Identifying mentorship opportunities can be difficult: the criteria for who can qualify as a mentee varies for each organisation, some requiring a young professional to be a certain age, ethnicity, or enrolled in education. Programmes often lack priority guidance from the leaders and mentors in charge, leaving mentees to develop self-initiatives skills through the creation of their own project or research. While this can be beneficial, removing mentorship opportunities limits the capacity for a YP to develop their career and professional networks. Potential mentors often exemplify a diffusion of responsibility, in which all of an organisation’s professionals are able to mentor; therefore, few take individual responsibility and do so. Limited infrastructure and ownership on the mentors’ side puts the onus on YPs to find mentorship, a difficult task considering the other barriers of inaccessibility and tokenisation previously mentioned. A lack of mentorship structure also limits the benefits of diverse mentoring, in which professionals at all career levels, from peers to senior professionals, participate. Therefore, further understanding of what constitutes a successful mentorship programme and how to develop, co-create, and sustain great mentors is needed [17].

## Knowledge gaps

The above listed barriers have proven difficult to overcome because of a cyclical lack of research and support. Indeed, inclusion by design is encouraged and reinforced when there are best practice examples. However, examples of relevant organisations and programmes are limited, as is the research literature regarding young professionals in the PHWF [9]. When a dearth of evidence exists, further elaboration or study is often not conducted. Furthermore, examples of seemingly routine recommendations, such as mentorship frameworks, do not exist in the literature, nor have the authors come across them in their own experiences as public health YPs. The level of diversity and heterogeneity of the PHWF itself has not been studied and valued, alluding to the larger context of a lack of infrastructure research within public health [18]. The existing structures and toolkits for change, such as those within mentoring programmes, rarely address larger structural changes or context adaptability [17].

## Opportunities and implications for policy and programming

Increasing the accessibility, integration, and mentorship of YPs within the PHWF has the potential for far reaching benefits for all stakeholders. Successful inclusion of YPs introduces opportunities for co-creation, intergenerational collaboration, and bi-directional learning [14]. Existing young professional programmes, such as those within associations like The Association of Schools of Public Health in the European Region (ASPHER), The World Federation of Public Health Associations (WFPHA), and The European Public Health Association (EUPHA), provide a strong basis upon which to act and engage with YPs. They should consider structural changes to increase accessibility, such as formalised mentorship programmes and remuneration schemas. YPs can bolster the capacity of the PHWF, contribute to build a less fragmented infrastructure, and be mutually beneficial for public health professionals at all points in their career. Bi-directional, intergenerational work will lead to better health programmes and outcomes [19]. YPs also have their own expert skills and relevant experiences that must be incorporated in conjunction with robust mentorship, so as to both include unique YP perspectives as well as grow their skills and networks. Such improvements can only be realised if sufficient physical and human resources are dedicated to ensuring the proper implementation and oversight of YP programs. Without proper infrastructure, resources, and mentors, YP programs can reinforce and perpetuate the very inequities that they aim to counteract, and subsequently result in the exclusion or loss of important voices [20]. To achieve a more successful, equitable, and sustainable PHWF through inclusion of YPs, organisations must remove monetary and resource barriers, increasing transparency and meaningful YP inclusion, and create more quality mentorship opportunities.

## Conclusion

COVID-19 has made clear the extreme needs of the PHWF. As societies discuss how to reshape the infrastructure of their systems, it is crucial that young professionals are genuinely involved. Previous attempts to incorporate YPs into the PHWF have wrestled with inaccessibility, tokenisation, and a lack of mentorship, leading to a loss of potential public health workers and an unrepresentative workforce which reinforces the systemic societal exclusion of diverse young people. These barriers must be addressed to build the sustainable, interdisciplinary, unified public health that is necessary for the future.

## Data Availability

Not applicable.
